# Pathways from climate change to emotional wellbeing: A qualitative study of Kenyan smallholder farmers living with HIV

**DOI:** 10.1371/journal.pgph.0002152

**Published:** 2023-07-25

**Authors:** Naomi S. Beyeler, Tammy M. Nicastro, Stanley Jawuoro, Gladys Odhiambo, Henry J. Whittle, Elizabeth A. Bukusi, Laura A. Schmidt, Sheri D. Weiser

**Affiliations:** 1 Institute for Global Health Sciences, University of California San Francisco, San Francisco, California, United States of America; 2 Department of Obstetrics, Gynecology & Reproductive Sciences, University of California San Francisco, San Francisco, California, United States of America; 3 Center for Microbiology Research, Kenya Medical Research Institute, Nairobi, Kenya; 4 Division of Psychiatry, University College London, London, United Kingdom; 5 Department of Global Health, University of Washington, Seattle, Washington, United States of America; 6 Department of Obstetrics and Gynecology, University of Washington, Seattle, Washington, United States of America; 7 Philip R. Lee Institute for Health Policy Studies and Department of Humanities and Social Sciences, School of Medicine, University of California San Francisco, San Francisco, California, United States of America; 8 Division of HIV, Infectious Diseases, and Global Medicine, Department of Medicine, University of California San Francisco, San Francisco, California, United States of America; Instytut Matki i Dziecka, POLAND

## Abstract

Climate change is associated with adverse mental and emotional health outcomes. Social and economic factors are well-known drivers of mental health, yet comparatively few studies examine the social and economic pathways through which climate change affects mental health. There is additionally a lack of research on climate change and mental health in sub-Saharan Africa. This qualitative study aimed to identify potential social and economic pathways through which climate change impacts mental and emotional wellbeing, focusing on a vulnerable population of Kenyan smallholder farmers living with HIV. We conducted in-depth, semi-structured interviews with forty participants to explore their experience of climate change. We used a thematic analytical approach. We find that among our study population of Kenyan smallholder farmers living with HIV, climate change is significantly affecting mental and emotional wellbeing. Respondents universally report some level of climate impact on emotional health including high degrees of stress; fear and concern about the future; and sadness, worry, and anxiety from losing one’s home, farm, occupation, or ability to support their family. Climate-related economic insecurity is a main driver of emotional distress. Widespread economic insecurity disrupts systems of communal and family support, which is an additional driver of worsening mental and emotional health. Our study finds that individual adaptive strategies used by farmers in the face of economic and social volatility can deepen economic insecurity and are likely insufficient to protect mental health. Finally, we find that agricultural policies can worsen economic insecurity and other mental health risk factors. Our proposed conceptual model of economic and social pathways relevant for mental health can inform future studies of vulnerable populations and inform health system and policy responses to protect health in a changing climate.

## Introduction

Climate change adversely impacts a wide range of health outcomes [1–3], with growing concern for mental health and emotional wellbeing [4, 5]. Studies link climate change to anxiety, depression, post-traumatic stress disorder, mood disorders, and suicidality [6, 7]. Many studies have focused on how discrete climate risks impact specific mental health outcomes [8, 9]. However, there a growing evidence pointing to the need for research on cumulative impacts of climate change and the ways in which those impacts affect mental health and wellbeing [10–12], which could range from trauma, to climate effects on livelihood and culture, to hopelessness and fear of the future [13–17].

Current literature on climate change and mental health emphasizes concepts of eco-anxiety [15], ecological grief [18], and solastalgia, which focus on the mental health impacts arising from ecological loss and the attachment to ecological places and resources [19, 20]. Climate vulnerability is shaped by social and economic factors that affect exposure to climate hazards and access to resources for resilience and risk mitigation [17, 21]. Yet comparatively few studies examine the social and economic pathways through which climate change affects mental health [22], even as these social determinants–including economic insecurity, job insecurity and sense of control over one’s employment, social cohesion, and family support–are well-documented drivers of mental health across the life course [23–27]. These social and economic factors amplify the health risks and adverse health outcomes caused by climate change [17, 28].

Mental health impacts of climate change are disproportionately experienced by communities that depend on environmental resources for economic security and cultural wellbeing, including Indigenous communities and farmers, as well as by people in low- and middle-income countries [5, 6, 18, 29]. However, research on climate change and mental health is largely based in high-income countries [8, 30], and while there is growing research in low- and middle-income countries [14, 31, 32] and Indigenous communities [33], little focuses on sub-Saharan Africa [8].

The objective of this study was to identify the potential social and economic pathways by which climatic changes impact mental and emotional wellbeing, focusing on an especially vulnerable population of smallholder farmers in Kenya living with HIV. Climate change is adversely affecting agriculture and food production, with harmful impacts on the economic and food security of farmers [34–36]. For them, climate change and the climate adaptation strategies they adopt are playing out in the context of largescale economic and agricultural changes in rural economies [37] that also have implications for mental health. Climate change–and drought specifically–is associated with HIV transmission and outcomes, as people living with HIV face higher risk of poverty, food insecurity, and other compounding vulnerabilities that make it harder to adapt to climate impacts and that increase HIV risk [38–40]. People living with HIV are also at risk of poorer mental health outcomes [41, 42].

For this study, we took advantage of the infrastructure of a larger study of Kenyan smallholder farmers living with HIV, described below, to conduct semi-structured interviews that explore how climate change shapes the mental and emotional wellbeing in communities already experiencing the day-to-day impacts of climate change. Drawing upon this case study, we propose a conceptual model for future studies of vulnerable populations in low- and middle-income countries and outline potential applications of this knowledge for public health programs, policy, and research.

## Methods

### Ethics statement

Ethical approval for the study was obtained from the review boards of the University of California San Francisco and the Kenya Medical Research Institute. All participants provided written informed consent.

### Research context

This is a qualitative research study located in Kisumu, Homa Bay, and Migori counties in western Kenya. This study is a sub-study of the *Shamba Maisha* cluster-randomized controlled trial (RCT) (NCT02815579): a 16-site study testing the effectiveness of an agricultural and finance intervention to improve health outcomes among farmers living with HIV. The *Shamba Maisha* intervention has been described in detail in other publications; intervention measures included provision of a non-electric water pump, a small loan for the purchase of farm supplies, and training on farming and financial management practices [43, 44]. Data collection for this study took place part-way through implementation of the *Shamba Maisha* study and included participants in both the control and intervention arms. Interviews were not designed to be evaluative of the impact of the intervention on mental health, but rather focused on the mechanisms through which climate change impacted mental health in a population highly vulnerable to adverse mental health outcomes. The impacts of the intervention on mental and physical health are presented in a separate publication of the *Shamba Maisha* RCT study results [45].

### Research setting and population

The East Africa region, and Kenya, is a climate vulnerable region experiencing climatic changes such as warming temperatures and extreme rainfall events [46, 47]. Climate-related morbidity and mortality are also projected to increase [48]. Climate change is projected to significantly reduce yields of Kenya’s major staple crops, contributing to food insecurity and malnutrition [49, 50]. Food insecurity is a pathway by which climate change can impact mental health [22, 33, 51]. In rural Kenya, a large share of the population relies on agriculture as a primary source of food and income [52]. There are 1.5 million people living with HIV in Kenya [53], and HIV positive households face significantly higher rates of food insecurity [54]. Farmers [15] and people living with HIV [42] are distinctly susceptible to adverse mental health outcomes.

### Study population

We purposively selected forty *Shamba Maisha* participants for enrollment in this study to include equal numbers of men and women, as well as control and intervention participants across the three study counties. *Shamba Maisha* project coordinators managed participant selection at study clinics; study research assistants conducted study enrollment and obtained written informed consent from all participants prior to participation. Eligibility criteria for the parent study included (a) living with HIV and receiving antiretroviral therapy (ART), (b) experiencing moderate to severe food insecurity or malnutrition at time of study enrollment (defined as BMI <18.5, and measured using the Household Food Insecurity Access Scale), (c) being 18 years or older, and (d) having access to farmland and surface water. Eligibility criteria for this study additionally included participation in the parent study for a minimum of one year.

### Data collection

Two research assistants, trained by the study team in qualitative interviewing and use of the interview guides, conducted in-depth interviews with participants. Interviews, conducted in Luo or Swahili, took place in study clinics, participants’ homes, or participants’ farms, depending on participant preference. We piloted interview guides in July and August of 2018 and collected data from September 2018 to February 2019. Throughout the piloting and data collection phases, we held weekly meetings between the U.S. and Kenya-based researchers to discuss interview findings and modify interview guides to address emergent themes.

We developed semi-structured interview guides collaboratively with both the U.S.- and Kenya-based research teams. Interview guides covered the following topics: perceived climatic changes and their causes; experience with discrete weather events and long-term weather changes; perceived impact of climate change on agricultural practices and outputs, income and economic security, and health outcomes and behaviors; pathways through which climate change affects HIV and mental health; and gender differences in the experience of and response to climate change. Interviews lasted from 90–120 minutes. All individuals invited to participate participated in the interviews. Participants were paid approximately four USD and reimbursed for transportation if the interview took place outside of their home. Interviews were audio recorded, then simultaneously translated and transcribed into English by the research assistants.

### Data analysis

We utilized a thematic analysis approach [55, 56] to analyze the interview data. Prior to coding the interviews, we read the transcripts to familiarize ourselves with the data and develop initial concepts for future coding. All coding in this analysis was based on emergent codes. For the early coding analysis, we applied an open coding approach with two researchers (NB, TN) independently creating and applying codes to each piece of transcript text. Subsequently, four researchers from both the U.S. and Kenya (NB, TN, GO, SJ) double coded a subset of interviews to jointly describe and discuss codes and code definitions, and to ensure the coding scheme accounted for and reflected the local context. Discrepancies in coding were resolved through discussion among all coders. We then developed a codebook through discussion of emerging concepts and themes, and used it to re-code interview transcripts in a more focused manner. In the final analysis, one author (NB) re-read and re-coded transcripts focusing on the themes of personal and communal mental health and wellbeing, to generate a deeper understanding of the themes that make up the focus of this paper. Given the potential for participation in the intervention arm to influence farmers’ experience of climate change, for instance through access to greater economic and agricultural resources, we considered participant characteristics including HIV status and participation in the farming intervention, in our analysis. Following our analysis, we consolidated our findings into a preliminary conceptual framework to highlight the key mechanisms through which climate impacts affected participant mental health and how these mechanisms interrelate.

## Results

This study included forty participants between the ages of 23 and 58 years of age, half of whom were men and half women. Participants described four major pathways through which climate change contributed to the emotional health and wellbeing impacts they experienced. First, participants reported direct mental health impacts from losing their homes or farms in extreme weather events. Second, participants reported emotional distress due to growing economic insecurity, largely resulting from climate impacts on agriculture and in some cases exacerbated by agricultural and development policies. Third, participants described changes to community, family, and social cohesion resulting from this rising economic insecurity–changes which led to emotional stress and weakened emotional resilience. Finally, for a subset of participants, a shifting sense of personal and professional identity driven by economic, employment, and family stress contributed to a decline in their emotional wellbeing. Across these pathways, participants described both directly-experienced impacts, and anticipated impacts–those that were expected to occur. Both experienced and anticipated impacts contributed to emotional distress. Although participants in the intervention and control groups had access to different resources to manage climate impacts on agriculture, in accounting for study enrollment characteristics in our analysis we found that the mechanisms, or pathways, by which climate change impacted emotional wellbeing were similar among participants in both groups. Thus, in the subsequent sections we describe these common pathways from climate change to emotional wellbeing. This qualitative study was not designed to assess the impact of the intervention on mental and emotional health outcomes.

### Direct impacts of climate change on the mental and emotional well-being of Kenyan farmers

Respondents nearly universally reported experiencing some level of climate change impact on their mental and emotional wellbeing. As one respondent commented, "Those of us who are farming are the ones likely to bear the brunt of climate change" (Man, 48 years, Migori). All study respondents reported direct experiences of changing weather, including higher temperatures; more drought, extreme rain events, and flooding; changes in the timing of the rainy season; and increasing weather unpredictability; though not all participants attributed these changes to the broader global phenomena of climate change. Participants described feeling “stressed”, “depressed”, “annoyed”, “demoralized”, “discouraged,” and “hopeless” because of these changes. One respondent explained that “the weather disoriented me completely" (Man, 38 years, Kisumu). Another described the stress of coping with unpredictability:

"You plant with so much hope of a better and food secure future…Then suddenly it rains, and everything is destroyed. You will be forced to start from zero. You get so affected mentally. Your thinking is distorted. You worry endlessly…You cannot know what happiness is at these times." (Man, 37 years, Kisumu)

Nearly all participants in the study described experiences of major damage to their homes and farms due to climate-related extreme weather events, losses that caused emotional distress and grief. One respondent described the loss of his farm as “equated to the death of a loved one. It was very painful” (Man, 53 years, Kisumu). Another participant reflected on how these emotional impacts affected his physical health:

“All my work had been drained just like that. I was deflated…My heart was bleeding, and my body became weak…Every time I would go to the farm, I would remember the loss…I lost appetite completely. I was so stressed up and worried.” (Man, 52 years, Kisumu)

Many participants described anger at the losses experienced. One participant reflected on the loss of her home and farm in a flood saying: “I could have jailed the rain if it was a human being” (Woman, 43 years, Kisumu). Other respondents spoke to the toll this took on their mental and economic well-being over time:

“I felt so devastated. I was depressed to the extent that I fainted. I couldn’t believe the loss I had just incurred and the damage the rain had done…I have never healed from that experience. I cannot do anything when it is raining because I get extremely scared…Weather has made me very angry and traumatized.” (Woman, 38 years, Kisumu)

In addition to these direct experiences of climate change, participants described anticipated future changes. Participants reported anticipating that future would be “difficult”, “rough” and “worse”, and that there was “no expectation for things to get better” (Woman, 27 years, Homa Bay). These negative expectations and fears about the future were a significant source of anxiety and worry.

### Pathways through which climate change impacts mental wellbeing

In addition to the direct impacts of climate change on emotional wellbeing described above, we identified three social and economic pathways through which climate change contributed to participants’ emotional health and wellbeing. These included economic impacts, such as declining agricultural productivity and economic stability; impacts to community social cohesion, such as declining social support; and impacts to personal identity, such as changes in professional and family role.

### Climate change erodes economic security of smallholder farmers

Economic insecurity was a major reported driver of participants’ mental and emotional distress. Participants described increasing economic insecurity resulting from direct climate impacts on agriculture, including declining agricultural productivity, property and crop losses. and the income unpredictability and instability that resulted. Climate disasters, such as unseasonable rain and flooding, destroyed farms and acutely impacted harvests. Participants “depend on farming” for their food and income; climate-related farm losses thus impacted their economic and food security. Many participants described experiencing significant declines in the productivity of their farms, sharing memories like this: “Floods came in April and all the maize, beans, and other cereals I had planted were swept away…There was nothing to harvest. The floods took all that we had worked for” (Man, 38 years, Kisumu).

Long-term climatic changes, such as drought and changing rainfall patterns also reduced farm productivity. As one participant described: “Droughts are more severe and frequent nowadays…We have no option but to sell our cattle at a throw away price. Nobody wants to be associated with such heavy losses" (Man, 27 years, Kisumu). Participants perceived these impacts to be widespread. One described: “There was a time when drought destroyed all the crops I had planted…This happens quite frequently, and to most farmers across this vast region” (Man, 23 years, Migori).

Many described immediate economic hardship following climate events. One participant described how a single night of heavy rainfall destroyed her home and crops and “took me back to poverty” (Woman, 38 years, Kisumu). Other respondents described how longer-term declines in farm yields led to long-term economic stress; “Income from the farm has greatly reduced, and this is because of the direct effect of the weather. We have suffered so much in our farming journeys” (Man, 45 years, Kisumu).

Economic losses led to extreme stress as a result both of the experienced disruptions to their livelihoods, and the anticipation that these experiences would continue or worsen in the future. As one respondent said "I had so many worries because I depended on the returns from my farm…Where was I going to get school fees? How was I going to feed my family?” (Woman, 47 years, Migori). Another stated: “Famine and poverty have pushed us to the wall” (Man, 58 years, Kisumu). Thus, not only did immediate economic stress and insecurity undermine emotional wellbeing, but also worry and fear about their expected economic prospects and their ability to provide for their families contributed to emotional distress independent of direct economic losses. In the words of one participant: “Being wealthy as a result of hard work is no longer a working narrative” (Man, 58 years, Kisumu).

Participants universally reported adapting their farming practices to respond to experienced and expected weather changes and to mitigate future losses. Participants described that climate change caused new farming challenges including pests and plant diseases, declining soil quality, and declining productivity of traditional crops. This necessitated a transition from “the practice our grandmothers taught us” (Man, 37 years, Kisumu) to more input-intensive farming techniques. Many respondents reported expanding the use of pesticides, fertilizers, and non-indigenous hybrid seeds.

However, participants described varying success in managing climatic changes and buffering economic stability. For some, these strategies made farming “more costly” and at times reinforced participants’ financial precarity and thus economic stress–and thus were actually maladaptive from a health perspective. As one participant said: “The amount of money we use in farming has tremendously increased…In the past you could farm and have plenty of harvest at zero costs. Now, I must buy fertilizer and seeds" (Man, 37 years, Migori). Often, as a result of adaptive strategies being insufficient to mitigate climate-related farm losses, those same strategies amplified economic insecurity. One participant described: "The capital we invest in the farm is much more than what we get from our farms" (Man, 42 years, Kisumu).

At the same time, as participants perceived climate change to make farming a less stable livelihood, some participants additionally perceived that broader land use and economic changes were making it harder for smallholder farmers to cope with climate change and deepened economic insecurity. For instance, several participants reported that the growth of large-scale commercial agriculture reduced access to land, leaving "very small pieces of land that cannot produce enough food" (Man, 37 years, Migori). Participants perceived this to affect economic and food security, as related by one: "All those commercial rice fields you saw were places where sorghum and millet were grown…So, there is a lot of famine” (Man, 58 years, Kisumu). Some participants also reported that changes in agricultural policies and markets were affecting their ability to earn income from farm products. Several respondents described local markets “over-flooded” with imported products, such that even when their harvests were strong, “the market was bad” and “prices fell greatly” (Man, 53 years, Kisumu). Another participant remarked: “The [local] market is flooded with imported rice…It outstrips demand…we sell [our] rice at a throwaway price" (Man, 58 years, Kisumu). Participants perceived these policy decisions as further straining their economic security, intersecting with and compounding the impacts of climate change on their agricultural practices, and consequently contributing to the emotional distress arising from economic factors.

### Economic insecurity undermines community support systems

Participants described how the economic impacts of climate change also affected the social drivers of emotional health at the community level. The widespread economic insecurity experienced by participants, together with an increase in perceived economic and food scarcity, disrupted community social cohesion by interrupting systems of communal support and mutual aid that historically buffered individual economic losses. This was an additional driver of worsening mental and emotional outcomes for some participants. Participants noted that those smaller harvests reduced community food availability: "In the past we had so much yield that we had to store in large granaries that were built outside our houses. Right now, you can walk for thousands of miles before seeing just one such granary." (Man, 37 years, Kisumu). As food insecurity grew, respondents described a shift in the culture of food sharing; "in earlier days you would walk into someone’s granary and take grains enough for your use, for free. Nowadays no one has harvest" (Man, 52 years, Kisumu). Another respondent said: “Nobody is giving out food for free. There is scarcity everywhere” (Man, 45 years, Kisumu).

As with economic impacts, both experienced and expected changes in social cohesion affected mental health. Some respondents perceived a reduced willingness among community members to support each other, reporting that people were “more individualistic”, “selfish”, and “concentrate only on [their] family”. One participant reflected: “Climate change and hard economic times have barred people from being as generous as they would want” (Man, 34 years, Migori), while others highlighted tension in balancing their desire to support others with the reality of scarcity:”The urge to give you some small tin will be there, yes, but I will have to reflect on how this might affect me and my family and then definitely I will tell you ‘No’” (Man, 50 years, Kisumu). The loss of social support systems contributed to emotional distress, as participants felt they had to rely on themselves even in times of need. Perceived lack of community support affected emotional health even when that support was not tested. As one woman described: “I would rather sleep hungry than beg my neighbors…I do not want that kind of shame” (Woman, 45 years, Migori).

### Economic insecurity affects personal identity

Finally, economic loss and insecurity also impacted emotional wellbeing by threatening participants’ personal expectations and sense of identity as caregivers and farmers, affecting individual level social drivers of poor mental health. This pathway to emotional stress was primarily discussed by men. Some participants reported a high degree of emotional distress when they were unable to provide for their family, challenging expectations they had for themselves as family head and primary household earner. As one participant described:

“[My] children wanted food which I couldn’t afford…It appeared to them that I hated them and did not want to provide as I should. This brought me a lot of mental torture. I wanted to give them the very best, but I couldn’t afford it…As a father, when you cannot provide for your children, it degenerates into a mental case.” (Man, 36 years, Kisumu)

Emotional distress from the inability to meet one’s personal expectations of their role as a caregiver could result from the direct experience of being unable to provide, as described above, or from the anticipated fear of future failure or declining social status. For instance, one participant described: "Your kids will constantly view you as a failure…You feel you cannot provide…The respect I expect from my wife and children won’t be given. I am almost useless " (Man, 52 years, Kisumu). These real and awaited changes in family cohesion created another potential stressor on participants’ emotional wellbeing.

A second disruption to participants’ sense of identity resulted from changes in agricultural work that affected the experience of farming, sense of control over their labor, and level of satisfaction in the profession. These changes could result in direct challenges to their farming identity, and consequently emotional distress. Some respondents reported taking on other jobs to supplement their income. This was another source of emotional stress, particularly for men, as having to work for others was viewed as demeaning: “I had gotten used to being my own boss. Now I had to start working at the mercy of other people…They deal with you the way they want. You are scolded like a kid in front of people” (Man, 26 years, Kisumu). Several men described embarrassment, disappointment, or loss of dignity at not being able to farm. One said: “You can’t even call me a farmer. I am a consumer. I live hand to mouth” (Man, 58 years, Kisumu). Others described how farming itself was increasingly viewed as a profession that could not sustain a person economically; in the words of a respondent: "[Farming] has been left to…those perceived to be poor" (Man, 45 years, Kisumu). The redefinition of farmers’ perceived role and position economically and socially was a source of emotional distress for these participants.

Distress could also result from anticipated threats to identity. As one respondent described: “You may have the [farming] knowledge, but the unpredictable weather pattern renders you useless” (Woman, 31 years, Migori). Unpredictability and repeated losses led some participants to feel a sense of futility about the future of agriculture. As one respondent asked: "Why plant crops only for the sun to scorch? Why plant crops for the floods to wash away? You cannot predict the weather, it either rains too much or there is too much drought" (Man, 42 years, Kisumu).

### Proposed conceptual framework

**Fig 1:**
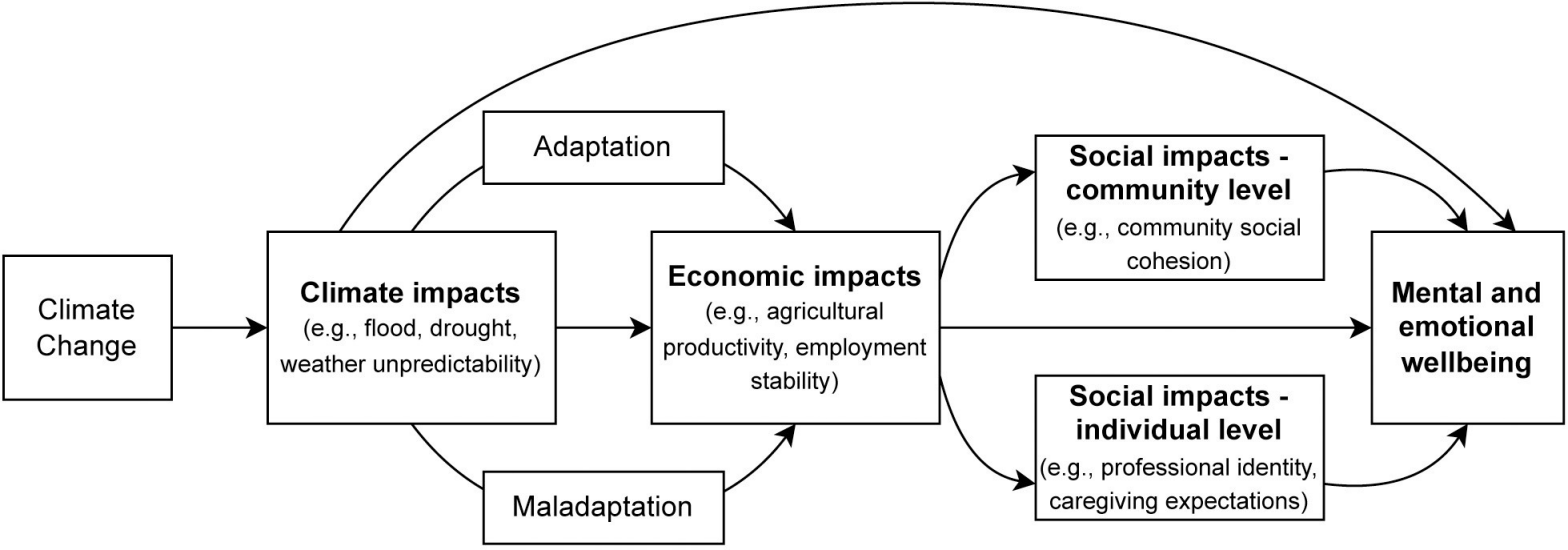
Simplified conceptual model of social and economic pathways linking climate change to emotional wellbeing.

Based on our analysis presented above, we consolidated our findings into a preliminary conceptual framework to capture the economic and social pathways by which climate change can shape mental and emotional wellbeing, and how these pathways are interrelated (Fig 1). This framework highlights the primary mechanisms through which we found climate change to affect emotional wellbeing. Climate impacts directly affect emotional health through experienced disruption, such as loss of a farm, and worry about the future. Climate impacts also drive economic shocks directly and shape economic security through the responses adopted by farmers, which can be either adaptive (improve resilience) or maladaptive (create further strain). Economic insecurity is a predominant mechanism of emotional distress. Finally, economic disruption creates social disruption both at the individual and community level, which in turn can each contribute to worsening emotional health. Across each of these domains, the arrows depicted in the figure, as described in the text above, can represent both directly experienced and anticipated or perceived potential impacts.

## Discussion

Climate change is having significant, cumulative effects on the mental and emotional wellbeing of Kenyan smallholder farmers living with HIV. As this population is especially vulnerable to both climate change impacts and poor mental health outcomes, it is thus an important community in which to develop a framework of the intersecting pathways between climate change and mental wellbeing. Emotional impacts experienced by participants included high degrees of stress, fear and concern about the future, and sense of sadness, worry, and anxiety stemming from the experience of losing one’s home or farm, or from a changing sense of personal and professional identity. These impacts on participants’ mental and emotional wellbeing were mediated by profound changes in the social and economic determinants of mental health, including increasing economic and food insecurity, declining status and economic viability of agricultural work, and the fragmentation of sources of resilience such as social cohesion.

Drawing upon these findings, we proposed a preliminary conceptual framework that includes four primary social and economic pathways that link climate change to adverse mental and emotional health, based on the lived experience of one highly vulnerable population (Fig 1). Studies in other countries have focused on discrete mental health outcomes (e.g., depression) or have largely captured these impacts using concepts such as eco-anxiety and ecological grief. Participants in this study described a wide range of impacts on their mental and emotional wellbeing from more general feelings of loss and sadness to more extreme experiences of emotional distress. While some participants in our study described feelings of eco-anxiety and grief, their predominant experiences were better captured as stress, worry, and identity displacement, experienced in terms of the social and economic changes wrought by climate change.

Our study found that individual adaptive strategies are likely insufficient to protect mental health in a rapidly changing climate. The psychology literature on coping identifies three broad categories of coping strategies: problem-focused–those that aim to directly change the stressor; social–those that draw on community and social support; and emotional–those that aim to manage personal emotional responses to the stressor [57]. This study shows how climate change is creating new stressors while also limiting many of these coping strategies. We find that, though farmers were proactively adapting their agricultural practices in the face of climate and economic volatility (i.e., problem-focused coping), often these strategies were insufficient to manage the level of disruption to their lives and their livelihoods, or to bolster the economic viability of small farms. Indeed, in some cases these strategies could exacerbate economic precariousness. These results suggest that these approaches, which are widely used by smallholder farmers in Kenya [58–64], may not be effective in buffering farmers from mental health stressors and poor mental and emotional wellbeing and may actually be maladaptive.

We additionally found that social coping strategies were increasingly limited due to the economic impacts of climate change. Participants experienced and anticipated changes in community trust, family and social cohesion, and emotional and economic support systems that traditionally buffer and protect from changing social and economic determinants. These social and cultural factors, identified in the social determinants of health literature as particularly important for mental health resilience [23], were being weakened as a result of climate change. Emotion-focused coping strategies may also be limited as emotional resilience declined in the face of growing stressors placed on individuals’ personal and professional sense of identity. Finally, across all pathways, we found that both directly-experienced and anticipated changes contributed to growing emotional distress, aligning with a broader psychology literature highlighting the importance of both lived and anticipated experience for mental health [65, 66]. This may be particularly important in the context of climate change, as, even if people are able to effectively adapt to and cope with current climate change, mental distress from anticipated impacts may grow as awareness of the directionality of future climate change increases.

We found that the socially and economically mediated experiences of climate change may vary by gender. Men more often emphasized stressors associated with the inability to meet gendered expectations to provide for their families, and with their shifting identity as farmers. While all participants experienced economic stressors, several men particularly emphasized the viability of farming itself and the identity changes that accompanied their challenges to succeed in this livelihood including their changing role and status within the family–something not discussed by women participants.

These findings, on the constraints individuals experience in adaptation and coping strategies, suggest that community and policy interventions are likely needed to adequately protect emotional and mental health in the face of climate change. This aligns with the broader adaptation literature research suggesting that individual level adaptation strategies can at times increase social risks and vulnerabilities [67–70]. However, an unexpected finding in our study was that agricultural and development policies were a concern for some smallholder farmers, with some policies perceived by participants to undermine their economic security, at a time when climate impacts were already straining their agricultural practices and income. Understanding the policy context is thus relevant for mental health, as this can contribute to economic insecurity–a primary driver of emotional distress.

There are several important limitations to the current study. The study was embedded within a larger trial testing the impact of an agricultural and finance support intervention on HIV health. Therefore, the participant sample is limited to individuals who are living with HIV and includes participants in an agricultural intervention that could impact their food and economic insecurity–factors which are themselves deeply connected to mental health. This may limit the external validity and generalizability of the results. For those participants in the intervention arm of the study, access to the training and financial support could affect their experience of climate change, for instance by enhancing access to resources that could bolster their awareness of and resilience to climate threats. This in turn could shape the relationship between climate change and mental health for these participants. To address this limitation, we considered the enrollment characteristics of participants throughout our analysis, finding that the pathways were similar across control and intervention groups. The interviews, conducted midway through the intervention period, were not designed to evaluate the impact of the intervention on climate resilience or mental health outcomes, but, rather to explore the mechanisms by which climate change and mental wellbeing are related in this population. This study found several emergent themes, such as the importance of agricultural and development policies to mental health pathways, on which further data collection was not possible within the study and should be investigated more fully. Additionally, the study was limited to interviews with farmers engaged in the *Shamba Maisha* trial and did not include other stakeholders such as local government or health care system representatives who could speak to the policy response to climate change and mental health.

Despite these limitations, our findings point to several important areas for future research and intervention in the growing field of climate change and mental health. First, future research could explore gendered pathways through which climate change shapes mental health. As climate adaptive strategies also differ by gender [71], this investigation should include understanding how various adaptation strategies protect or harm mental health across genders. Future research could also explore strategies to strengthen mental and emotional resilience for vulnerable populations, which could include community and health system approaches such as leveraging existing health care services to expand and integrate mental health care. However, while such mental health supports are important, our study highlights the fact that efforts to improve global mental health must simultaneously address climate change and the social and economic determinants of mental and emotional wellbeing, and that clinical mental health interventions alone are unlikely to protect mental and emotional wellbeing in a rapidly changing climate. Thus, future research should focus on such solutions. Studies could include investigations of adaptation solutions: to understand whether they would be responsive to vulnerabilities that may result from local coping practices, what impact they might have on mental health risks, and in what ways they could be strengthened to foster more successful adaptation and to protect against the mental health risks of climate change. A multisectoral policy approach, engaging health, environment, and economic sectors, is needed to buffer the mental health risks of climate change–yet these policy issues are infrequently discussed in the mental health literature. Given the centrality of economic pathways to mental wellbeing in this study, this multisectoral policy approach should be an additional focus for future research.

This study highlights the profound emotional distress experienced in this climate vulnerable community. It advances the literature on climate change and mental health by describing the importance of economic and social determinants in mediating the relationship between climate change and mental health, and by elevating the importance of policy solutions as both contributors to growing emotional distress and as essential responses in the face of adaptation constraints. Further exploration of potential economic and social factors is therefore important, to understand how climatic changes–and the adaptation and coping responses people turn to in response–shape the mental and emotional wellbeing of climate vulnerable communities.

## Supporting information

S1 Text Inclusivity in global research checklist. (DOCX)
